# Tailoring thermal conductivity via three-dimensional porous alumina

**DOI:** 10.1038/srep38595

**Published:** 2016-12-09

**Authors:** Begoña Abad, Jon Maiz, Alejandra Ruiz-Clavijo, Olga Caballero-Calero, Marisol Martin-Gonzalez

**Affiliations:** 1 IMM-Instituto de Microelectrónica de Madrid (CNM-CSIC), Isaac Newton 8, PTM, E-28760 Tres Cantos, Madrid, Spain

## Abstract

Three-dimensional anodic alumina templates (3D-AAO) are an astonishing framework with open highly ordered three-dimensional skeleton structures. Since these templates are architecturally different from conventional solids or porous templates, they teem with opportunities for engineering thermal properties. By establishing the mechanisms of heat transfer in these frameworks, we aim to create materials with tailored thermal properties. The effective thermal conductivity of an empty 3D-AAO membrane was measured. As the effective medium theory was not valid to extract the skeletal thermal conductivity of 3D-AAO, a simple 3D thermal conduction model was developed, based on a mixed series and parallel thermal resistor circuit, giving a skeletal thermal conductivity value of approximately 1.25 W·m^−1^·K^−1^, which matches the value of the ordinary AAO membranes prepared from the same acid solution. The effect of different filler materials as well as the variation of the number of transversal nanochannels and the length of the 3D-AAO membrane in the effective thermal conductivity of the composite was studied. Finally, the thermal conductivity of two 3D-AAO membranes filled with cobalt and bismuth telluride was also measured, which was in good agreement with the thermal model predictions. Therefore, this work proved this structure as a powerful approach to tailor thermal properties.

Three-dimensional anodic alumina templates (3D-AAO) are an exciting new form of network^1^, which are composed by a geometric space-frame of interconnected nanoholes that can be filled with other materials. The open space-frame structure of 3D-AAO membranes provides them with some notable properties. For instance, they can act as a host to combinations of functional materials within the interstices of the framework, which can be used to design materials with exotic properties. Ordinary AAO membranes stand for thick-film-like with a close hexagonal packed structure and longitudinal pores perfectly parallel whose pore diameter can be tuned by the fabrication conditions from 8 nm to 530 nm^2^,^3^,^4^. 3D-AAO structure has been recently developed which allows scientists to study different kinds of physical phenomena when it is filled by diverse materials^1^,^5^. These structures consist of longitudinal pores as the regular AAO membranes with the incorporation of transversal pores interconnected so that a highly ordered 3D network is established with a hexagonal arrangement in both longitudinal and transversal directions. Moreover, alumina presents a high mechanical, chemical and thermal stability which made this material suitable for many kinds of investigations^6^,^7^,^8^.

To date, optical phenomena have been studied when these 3D alumina structures are filled by materials like polymers or bismuth telluride, showing a photonic crystal behavior^1^,^5^. However, there is a lack of investigations regarding the thermal properties of these structures. They can be used not only as suitable building blocks for thermal engineering by introducing different filler materials into the framework but also to study the influence of the nano-architecture in the heat transport of the filler material itself. In this sense, the study of the thermal conductivity in this structure is certainly attractive, since the low dimension of the interconnected channels can induce a reduction in the thermal conductivity of the filler material by phonon scattering processes. This reduction can be a promising approach in fields like thermoelectricity since the efficiency of the thermoelectric materials is inversely related with their thermal conductivity^9^. As seen previously, the thermal conductivity of the alumina membranes does not suffer any decrease when reducing the dimensions since anodic aluminum oxide is an amorphous material so that the phonon mean free path is in the order of the interatomic distance and hence, the heat conduction is not affected by a reduction in the dimensions^10^. In order to study the thermal conductivity of the filler materials, it is necessary not only to know the porosity and the skeletal thermal conductivity of the alumina membrane itself but also to establish a thermal model which allows the determination of the thermal conductivity of the filler material. In the case of regular AAO membranes, the effective medium theory can be applied since the membrane can be approximated to two thermal resistors in parallel and the thermal conductivity is given by the following expression:


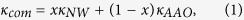


where *κ*_*com*_ is the effective thermal conductivity of the composite (also known as composite thermal conductivity), *x* is the porosity, *κ*_*NW*_ is the thermal conductivity of a single nanowire and *κ*_*AAO*_ is the AAO skeletal thermal conductivity. In the case of the 3D alumina, this model is not valid since this sample can be seen as a thermal resistor which combines resistors both in series and in parallel. Many works have dealt with similar difficulties before. Most of them approached the problem by utilizing sophisticated computational methods^11^,^12^. For instance, Toledo *et al*.^13^ extended the effective medium theory to incorporate anisotropic regular lattices aside of their connectivity structure which could be applied to the transport properties calculation of 3D structures with cubic symmetry. Hui *et al*.^14^ reported two methods to model the thermal conductivity of a graded composite film in both parallel and perpendicular directions to its surface. Other studies showed new heat transfer models by using a simple network of thermal resistors. More specifically, Staggs^15^ developed a 3D model which was used to calculate the thermal conductivity of alumina with spherical air inclusions inside. In this case, a considerable disagreement with experimental data when the porosity is higher than 25% was found. Therefore, a simple thermal heat transfer model with no advanced computational requirements for the complex 3D porous hexagonal ordered structures is required in order to design new promising materials.

In this work, the effective thermal conductivity of an empty 3D AAO membrane was measured by the photoacoustic technique, which has been previously established as a suitable technique to measure this type of structures^10^. For this measurement, a characterization of the density, specific heat, and porosity has been carried out since the thermal model used by this technique made use of these magnitudes in order to extract the effective thermal conductivity of the sample. Moreover, once the effective thermal conductivity was extracted, a thermal conduction model was developed in order to obtain the thermal conductivity of the skeletal 3D alumina membrane whose value is needed for future investigations of possible filler materials. This thermal model was used to study the influence of different filler materials and the geometric parameters of the 3D alumina (length and number of transversal nanochannels) which can be controlled during the fabrication process, demonstrating that the effective thermal conductivity of the whole composite can be tuned by these parameters and thus, making possible to control the heat flux across the structure depending on the application while keeping lateral conduction. Finally, the thermal conductivity of two 3D-AAO membranes filled with cobalt and bismuth telluride was measured to prove the validity of the thermal model.

## Results and Thermal Model

**Figure 1:**
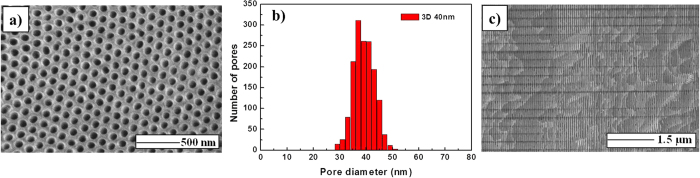
(**a**) Top-view (**b**) pore diameter histogram and (**c**) cross-section of the 3D AAO membrane.

The morphology of both top and transversal views of the three-dimensional nanostructure was characterized by Scanning Electron Microscopy (SEM) as SEM as shown in Fig. 1. The top view [Fig. 1(a)] shows a hexagonal close-packed arrangement of the pores. The pore diameter of the longitudinal pores was studied by a histogram as shown in Fig. 1(b), showing an average pore diameter of 40 nm as the pore size distribution indicates. Originally, pores of 25 nm were obtained from the sulfuric acid solution^10^ but a larger final pore diameter was obtained due to the widening process during the etching step. Figure 1(c) shows the cross-sectional view where the longitudinal pores along with the transversal nanochannels in the horizontal direction of the image can be appreciated, thus demonstrating the 3D nanochannel network formation as observed by Martin *et al*.^1^. Moreover, as explained in the ref. 1, the transversal nanochannels present also hexagonal symmetry. From Fig. 1(c) an average diameter in the vicinity of 30 nm and an interchannel distance of approximately 230 nm were measured for the transversal nanochannels. The number of nanochannels, *N*, is designed in the experiment which depends on the number of pulses selected in the software used for the second anodization process. In this work, 120 nanochannels were obtained. Moreover, both the top and bottom parts of the 3D alumina layer present around 1.2 μm without any transversal nanochannels which provide mechanical stability to the whole sample. Finally, the length perpendicular to the 3D membrane surface was found to be 33.4 μm.

**Figure 2:**
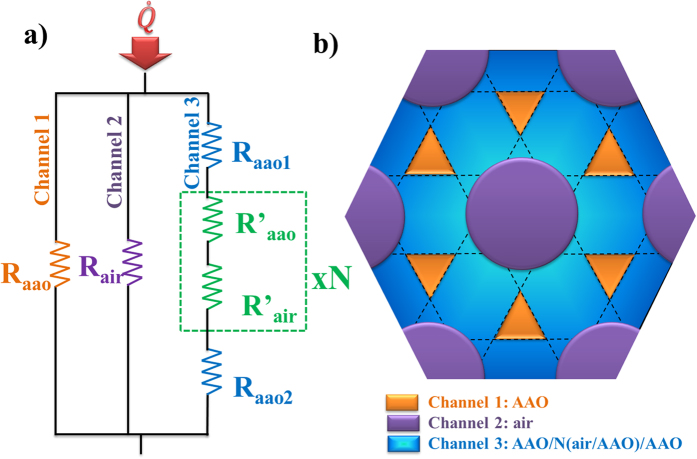
(**a**) 3D AAO sample thermal resistor model where 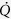 corresponds to the heat going through the samples and (**b**) top-view of a section with the transversal channels (dashed lines) of the 3D ordered AAO membrane.

In order to extract the composite thermal conductivity by the photoacoustic technique, it is necessary to characterize the density, porosity, and specific heat, showed in Table 1 (See measurement details in Supporting Information). The resulting effective thermal conductivity was very low, *ĸ*_*com*_ = 0.38 ± 0.06 W·m^−1^·K^−1^, compared with an ordinary AAO with longitudinal pores and much larger porosity, 58%, whose effective thermal conductivity resulted in *ĸ*_*com*_ = 0.64 W·m^−1^·K^−1 ^10. The explanation can be found in the thermal conduction model for each sample. In the case of the sample from ref. 10, the sample consists of a common AAO template with longitudinal pores. The heat conduction based on the Effective Medium Theory (EMT) can be understood as a thermal parallel resistor so that the heat flux can find a path to go through the cross-plane direction. However, in the case of 3D structure, the heat conduction model is more complicated since it involves a combination of parallel and series heat conduction. The EMT must be modified in order to obtain the skeletal thermal conductivity. If EMT is applied, an underestimated value of the skeletal thermal conductivity will be calculated. This value results in 0.62 W·m^−1^·K^−1^ when the overall porosity (40%) is considered. However, the 3D sample must be seen as a thermal resistor circuit as shown in Fig. 2(a). This circuit has three parallel components which are depicted in Fig. 2(b): two of them are common to the EMT, i.e., the AAO (*R*_*aao*_) and the pores (*R_air_*) contribution, but there is a new term which arises from the interconnected transversal channels. The last component is composed by several series resistances: the AAO on the top (*R_aao1_*) and bottom (*R_aao2_*) of the sample before and after the transversal channels start to appear located in the top and bottom parts of the membrane and the resistances associated with the channels (*R’ _aao_* and *R’ _air_*) which should be multiplied by *N*, being *N* the number of channels. It is worthy of note that this circuit is a simplified model which neglects the thermal contact resistances between longitudinal pores as the effective medium theory, but also neglects the thermal contact resistance between the air and the alumina in the transversal nanochannels as a first approximation.

With these assumptions, the mathematical model is derived from this circuit so that the total thermal resistance is given by this expression:


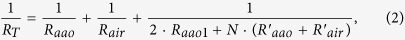


where each of the components is defined as:


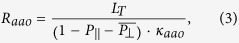



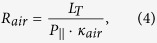



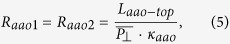



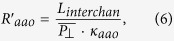



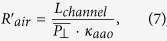


where *R*_*T*_ is the equivalent resistance of the whole system, *L*_*T*_ is the length the sample, *P*_||_ is the top-porosity associated with the longitudinal channels, *k_aao_* is the skeletal thermal conductivity of the AAO membrane, *k*_**air**_ is the air thermal conductivity, *L*_*aao−top*_ is the length at the top of the AAO membrane without transversal nanochannel, which is considered to be around the same as the length of the bottom of the AAO membrane without transversal nanochannels, 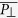 is the mean top-porosity corresponded to the transversal nanochannels, *L_interchan_* is the distance between two transversal nanochannels, and *L*_channel_ is the average pore diameter of these nanochannels.

If we substitute each magnitude in Eq. (2) we arrive at the following expression for the effective thermal conductivity:


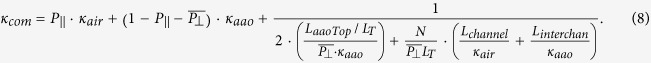


**Figure 3:**
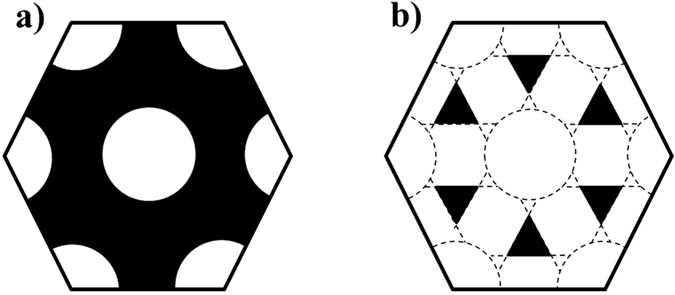
(**a**) Cross-section of the 3D AAO membrane of a region without transversal channels and (**b**) cross-section of the 3D AAO membrane of a region with the transversal channels. The black and white regions correspond to solid AAO membrane and air, respectively. Both sketches correspond with a hexagonal unit cell. All the dimensions have been taken into account from the SEM image: D_p_ = 40 nm; D_int_ = 66 nm; L_channel_ = 30 nm, where D_p_ is the pore diameter, D_int_ is the interpore distance and L_channel_ is the transversal channel size.

**Figure 4:**
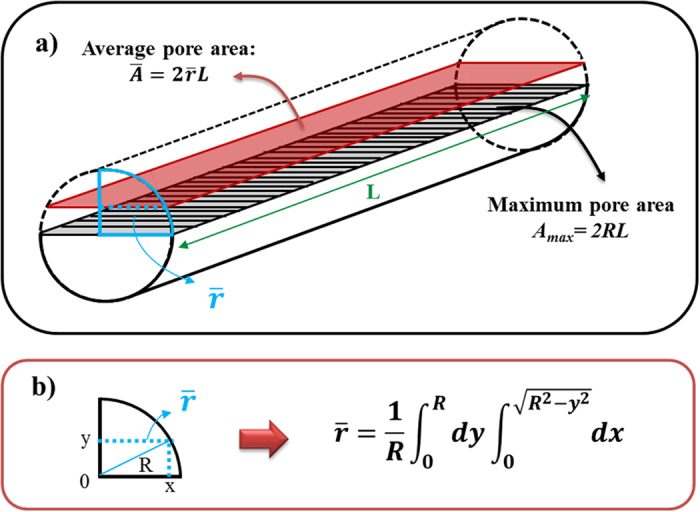
(**a**) Sketch of the transversal pores between longitudinal pores. The shadow area is the average area of the pore. (**b**) Calculation of the average radius which provides the average radius used to calculate the average area of the pore.

In order to calculate 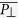, it is necessary to know the top-view porosity corresponding to the transversal nanochannels, 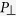. In Fig. 3, a top view of a hexagonal cell without (a) and with (b) transversal channels at full scale is depicted. An image analysis of both images was carried out, giving the associated porosities in each case. In order to verify that this analysis is valid for our sample, the same analysis corresponded to Fig. 3(a) is performed in five SEM images corresponding to the top view of a 3D sample, which provides a value of 31.8%. The analysis of the sketch of Fig. 3(a) gives a value of 33.8%. The difference between these two values can be considered within the error interval. This verification allows us to calculate the porosity of a cross-section of the 3D AAO structures with the transversal nanochannels by means of Fig. 3(b) since it is not possible to measure it by SEM images analysis. A value of 90.3% is obtained, which takes into account the transversal nanochannels along with their intersection with the longitudinal pores. Therefore, the transversal nanochannel porosity is 90.3−31.8 = 58.5%. That means that in a cross-sectional view with transversal nanochannels, 58.5% of air arises from transversal nanochannels. Nevertheless, this value corresponds to the cross-section of the pore where its area is maximized. Actually, there is a gradual porosity along the transversal channel, i.e., in the cross-plane direction as seen in Fig. 4(a). In order to take this effect into account, it is necessary to make some calculation which provides the mean transversal porosity.

The mean value of a continuous function is defined as:


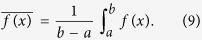


In our case, the function is given by the section of the transversal pores which are considered as cylinders (A = 2 R·L). The problem can be reduced to the calculation of the average radius of the cylinder as Fig. 4(b) depicts. The limit of integration of the *x* variable is calculated by the Pythagorean Theorem, so that the average pore area can be calculated as:


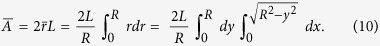


If the first integral is developed:


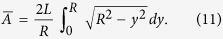


By integrating we finally derived the mean value of the cross-sectional area of the transversal pores:


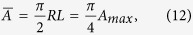


where *A*_*max*_ is the section area of the pore with radius *L*_*channel*_*/2* and *L* is the distance between two longitudinal pores, i.e., the wall thickness. The porosity is proportional to the area so that, the same relation can be found for 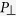. In this case, we are looking for the mean value of 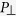 which is given by:


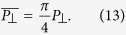


With this procedure, we finally obtain a value of 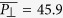
%.

Moreover, this model allows the calculation of the overall porosity of the 3D AAO membrane which is given by:


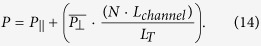


If values of Table 2 are taken into account, the 3D AAO membrane porosity is found to be in the vicinity of 37%, which is within the error interval of the porosity calculated by the BET technique, 40 ± 6%.

### Skeletal thermal conductivity calculation

The fixed values used in this model along with each information source are presented in Table 2. The top-porosity, 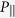, was obtained by SEM image analysis with the imageJ software. The air thermal conductivity was taken as a constant. The size of the channels was measured from the SEM images along with a calculation by taking into account that the number of channels, *N*, is a fixed quantity that is introduced into the software before the anodization process. The total length of the membrane is measured from the SEM images. Finally, the average porosity associated with the transversal channels, 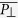. is obtained by averaging the porosities along the radius of the transversal pore, as explained above.

If the 3D model is employed, a value of 1.25 ± 0.25 W·m^−1^·K^−1^ is obtained for the skeletal 3D AAO membrane which matches the found values obtained for the AAO prepared in sulfuric acid solutions^10^. This result serves to prove the validity of the 3D thermal model which can be very useful to study the thermal conductivity of different filler materials inside the 3D AAO matrix. The simplicity of the resistor network used to describe the 3D AAO structure removes the necessity of sophisticated computational methods. Moreover, this model allows the calculation of the overall porosity by a simple image analysis and avoids the need of carrying out further BET experiments. Furthermore, there is no restriction regarding the thermal conductivity ratio between the filler material and the skeletal alumina as in the case of Nan *et al*.^16^ who found a deviation from the conventional effective medium approximation in a system formed by carbon nanotubes dispersed in a matrix when larger differences in their thermal conductivities are used. Moreover, this model allows the study of the effective thermal conductivity when varying the geometric features of the overall structure as explained below.

### Thermal conductivity engineering

Once the skeletal thermal conductivity of the 3D AAO template was extracted, a prediction of the composite thermal conductivity as a function of the number of nanochannels and the length of the AAO was carried out. For that aim, the associated term to the top and bottom part of the AAO without transversal channels is neglected, provided that their length is lower than the 25% of the whole membrane, so that the total length of the alumina would be given by:


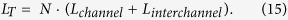


Moreover, it is necessary to impose a constraint condition which assumes that the total length of the nanochannels cannot be equal or overtake the total length of the AAO membrane. In the case that both lengths were equal, the interchannel distance would be zero, which does not make sense. Experimentally, the minimum interchannel distance that can be achieved for *L_channel_* = 30 nm is approximately 30 nm so that, the constraint condition expressed in micrometers would be:


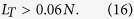


And the composite thermal conductivity can be expressed as a function of the *L*_*channel*_, *N* and *L*_*T*_ as:


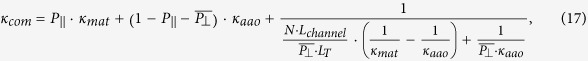


where, *κ*_*mat*_ is the thermal conductivity of the filler material. In this case, the previously calculated value of 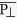 will be used so that it assumes *L_channel_* = 30 nm as a fixed quantity.

**Figure 5:**
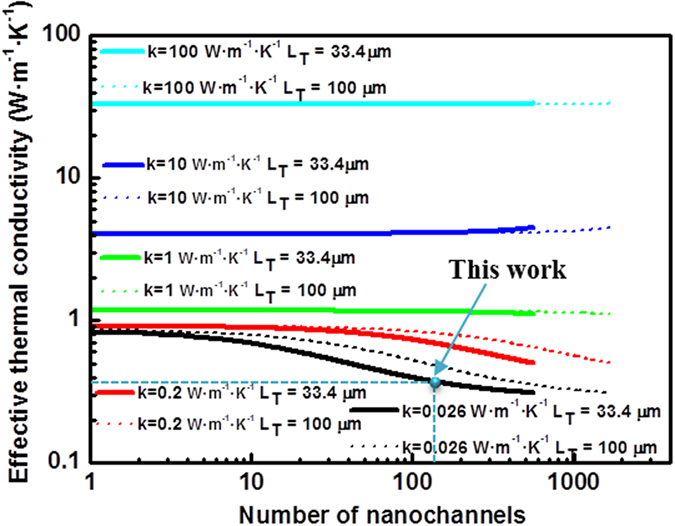
Effective thermal conductivity as a function of the number of the nanochannels which follows Eq. (17) for two different total 3D AAO membranes lengths and filler materials with different thermal conductivities, k = 0.026 W·m^−1^·K^−1^ (black line), k = 0.2 W·m^−1^·K^−1^ (red line), k = 1 W·m^−1^·K^−1^ (green line), k = 10 W·m^−1^·K^−1^ (dark blue line), k = 100 W·m^−1^·K^−1^ (light blue line).

Figure 5 shows the tendency of the effective thermal conductivity of two AAO membranes with different lengths for air and four filler materials with different thermal conductivities. It is straightforward to find out the effective medium theory when the number of nanochannels is *N* = *0* in Eq. 17. On the other hand, there is a limit on the number of nanochannels which is given by the constraint condition of Eq. (16). In Fig. 5 this limit is 556 and 1666 nanochannels for lengths of 33.4 μm and 100 μm, respectively. It is clearly seen that it is feasible to control the effective thermal conductivity of the whole composite by manipulating the number of nanochannels and the length of the alumina template, which directly implies a control of the interchannel distance since three magnitudes are correlated by means of the Eq. (15).

**Figure 6:**
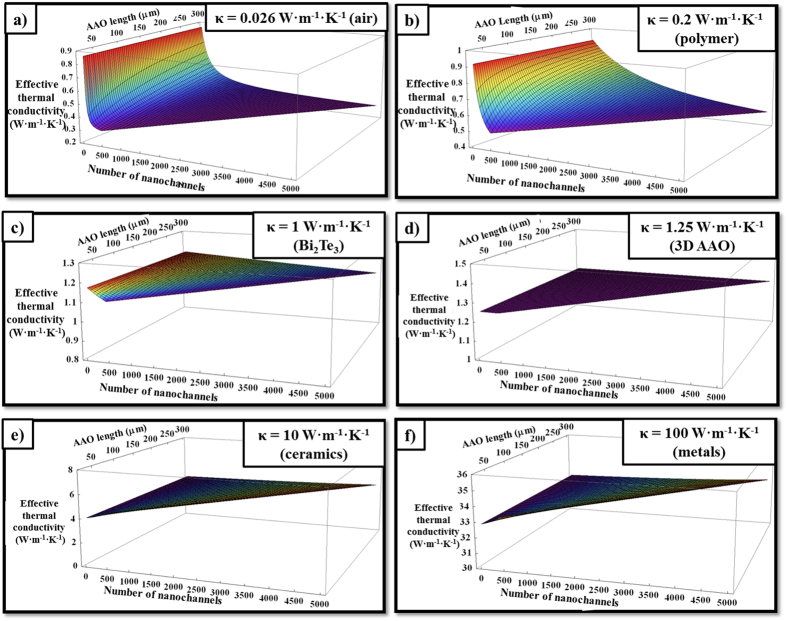
Effective thermal conductivity as a function of the number of nanochannels and the length of the 3D AAO membranes for filler materials with different thermal conductivities (**a**) 0.026 W·m^−1^·K^−1^ (air) (**b**) 0.2 W·m^−1^·K^−1^ (polymer) (**c**) 1 W·m^−1^·K^−1^ (Bismuth telluride), (**d**) 1.25 W·m^−1^·K^−1^ (3D AAO), (**e**) 10 W·m^−1^·K^−1^ and (**f**) 100 W·m^−1^·K^−1^.

However, it is important to study the effect of varying both the number of transversal nanochannels and the length of the 3D AAO structure in the effective thermal conductivity at the same time. Figure 6 shows this variation for the empty alumina matrix [Fig. 6(a)] and four different filler materials of different thermal conductivities: a polymer whose thermal conductivities are typically known as 0.2 W·m^−1^·K^−1^ (like PCDTBT^17^) [Fig. 6(b)] and materials whose thermal conductivity is in the order of 1 W·m^−1^·K^−1^ (like bismuth telluride^18^) [Fig. 6(c)], 10 W·m^−1^·K^−1^ (ceramics) [Fig. 6(e)] and 100 W·m^−1^·K^−1^ (metals) [Fig. 6(f)]. Finally, Fig. 6(d) corresponds to the effective thermal conductivity values calculated when a filler material with the same thermal conductivity of the alumina template is used, 1.25 W·m^−1^·K^−1^. As expected, the obtained value is the AAO membrane value and it is completely independent of the number of nanochannels and the thickness of the sample since it could be seen as a bulk material and, thus, corroborating the validity of Eq. (17).

Figure 6(a,b and c) show a reduction in the overall thermal conductivity when the filler material is less thermally conductive than the AAO membrane and the number of nanochannels is augmented, since increasing the number of nanochannels implies to increase the relative amount of filler material. An opposite trend can be seen when increasing the thickness of the alumina membrane for a fixed number of nanochannels; the effective thermal conductivity of the whole composite increases since the interchannel distance increases in such a way that the relative quantity of alumina in comparison with the filler material is increased. If the length tends to infinite, the effective thermal conductivity will reach the value corresponding to the EMT as it can be derived from Eq. (17). In the case of filler materials with higher thermal conductivity than that of the AAO membrane, the effective thermal conductivity tends to the effective thermal conductivity value corresponding to the regular AAO without transversal nanochannels, i.e., the EMT value, as shown in Fig. 6(e,f) where a great reduction of the effective thermal conductivity in comparison with the filler material itself is achieved, from 10 W·m^−1^·K^−1^ and 100 W·m^−1^·K^−1^ to 4 W·m^−1^·K^−1^ and 32 W·m^−1^·K^−1^, respectively. This value leans to be independent of the number of the nanochannels and the length since the thermal resistance of the filled nanochannels is much lower compared with the longitudinal filled pores.

### Thermal model validation: 3D AAO membranes filled with i) Cobalt and ii) Bismuth telluride

**Figure 7:**
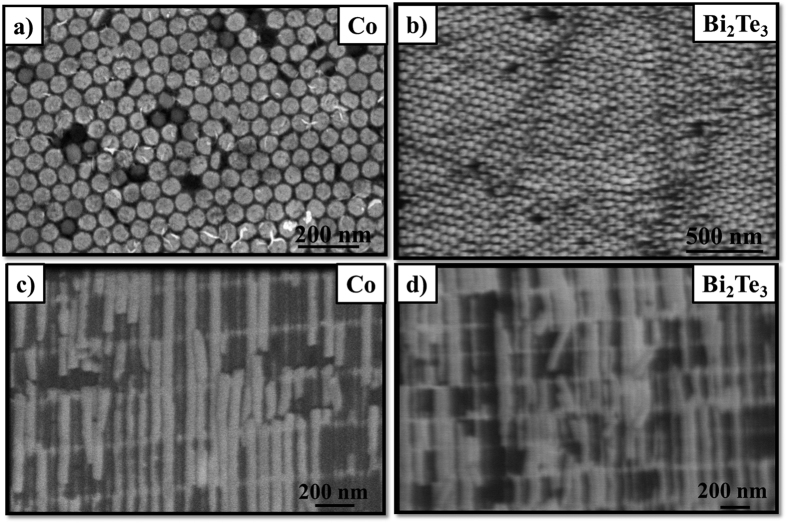
Top-view of (**a**) cobalt and (**b**) bismuth telluride and cross-section of the 3D membrane filled with (**c**) cobalt and (**d**) bismuth telluride.

In order to prove the validity of the thermal model developed for this structure, two different 3D AAO membranes, one filled with cobalt, whose thermal conductivity is 100 W·m^−1^K^−1 ^19, and the other filled with bismuth telluride (~1.7 W·m^−1^K^−1^) were measured. Both materials were grown by electrodeposition as explained in the Supporting information. Figure 7(a,b) shows the top view of the AAO membrane filled with cobalt and bismuth telluride, respectively, where the tips of the nanowires of around 55 nm can be distinguished. Moreover, it is important to note that the filling factor, i.e., the actual percentage of filled pores, is rather high. By analyzing several SEM images, a filling factor of 95% and 99% was calculated. Figure 7(c,d) shows a cross-sectional view of the cobalt and bismuth telluride samples where the filled transversal nanochannels can be observed.

The density and specific heat were calculated as explained in the Supporting Information. After carrying out several PA measurements of the sample on different regions, the effective thermal conductivity was found to be 69 ± 7 W·m^−1^K^−1^ and 0.75 ± 0.08 W·m^−1^K^−1^ for the cobalt and bismuth telluride samples, respectively. We used this value in order to calculate the thermal conductivity of the cobalt and bismuth telluride structures inside the matrix by means of the thermal model proposed, along with the values showed in Table 3. These values were mainly obtained by SEM images together with a porosity analysis similar to the one performed for the empty 3D AAO membrane. A cobalt value of 97 ± 37 W·m^−1^K^−1^ was calculated which was in excellent agreement with the theoretical value of bulk cobalt, 100 W·m^−1^K^−1^. In the case of the bismuth telluride, a value of 0.58 ± 0.22 W·m^−1^K^−1^ was calculated which is in good accordance with a very recent work which showed a great reduction in the thermal conductivity of Bi_2_Te_3_ when reducing the pore diameter due to an increment of phonon scattering. This work shows a thermal conductivity value of 0.72 ± 0.37 W·m^−1^K^−1^ for nanowires of 52 nm which is within the error uncertainty of our measurement. This last measurement confirms the capacity of the model to distinguish size effects. Also, both the thermal model and the experimental measurement reliability are confirmed for empty and filled alumina samples with an uncertainty of around 25–35%.

Moreover, a cobalt film prepared by electrodeposition onto a glass substrate covered with a gold layer was also measured in order to investigate if there is any influence on the material due to the electrodeposition process in comparison with the bulk material. A film of 40 μm was grown and measured by the PA technique, giving a cobalt value of 99 ± 10 W·m^−1^K^−1^ which is also in good agreement with the expected bulk value. The fittings used to extract both values, the film and the composite cobalt-filled 3D AAO membrane, are shown in the Supporting Information.

These 3D structures allow the tuning of the effective thermal conductivity of the composite by controlling the fabricating parameters, i.e., the thickness, the number of nanochannels and the interchannel distance of the 3D AAO membrane. In the low thermal conductivity regime of the filler materials, from air to polymer filler materials, variations up to 60% in the effective thermal conductivity can be achieved for a 3D AAO membrane filled with the same material just by tuning the referred parameters. Conversely, in the case of filler materials with higher thermal conductivities, this model demonstrates that it is possible to design a composite with a selected thermal conductivity by choosing the proper top porosity and the filler material. This structure is greatly advantageous since the three-dimensional framework ensures electrical conduction in the in-plane direction while reaching a thermal conductivity lower than that of the filler material itself, which could be promising in fields such as thermoelectricity. Besides, this model could be used to detect nanosize effects since any deviation of the values will be related to phonon boundary scattering.

## Conclusions

The effective thermal conductivity of an empty 3D anodic aluminum oxide membrane has been measured by the photoacoustic technique. The values of the density, specific heat and porosity of the sample have been characterized in order to carry out the data reduction needed by this technique. Moreover, a 3D thermal model based on a combination of a parallel and series thermal resistances was developed in order to obtain the skeletal thermal conductivity of the 3D ordered AAO membrane, 1.25 ± 0.25 W·m^−1^·K^−1^. This model was used to determine the influence of the number of transversal nanochannels as well as the thickness of the 3D AAO structure for empty and filled with materials of different thermal conductivities, showing a capability of tuning the effective thermal conductivity as a function of those magnitudes. The validation of the thermal model was proved by measuring two different 3D AAO membrane filled with cobalt and bismuth telluride, showing experimental values of thermal conductivity in very good agreement with predictions and therefore, the capability of tailoring thermal properties by this novel 3D structure.

## Methods

The porosity of the 3D AAO sample is composed by the longitudinal channels along with the transversal ones. The porosity associated with the longitudinal channels will be called top-porosity, 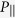, and can be calculated by means of the SEM image analysis by the imageJ® software. Several images from different zones of the sample were analyzed in order to reduce the uncertainty. However, the porosity related to the transversal channels cannot be measured by image analysis. The overall porosity was measured by using the BET technique for surface area calculation and the Barrett – Joyner – Halenda (BJH) method which provides information about the average pore size and pore volume of the sample (see Supporting information). The density and the specific heat were measured in order to extract the thermal conductivity of the 3D AAO membrane by means of the photoacoustic technique^20^ (see Supporting Information for further details).

## Additional Information

**How to cite this article**: Abad, B. *et al*. Tailoring thermal conductivity via three-dimensional porous alumina. *Sci. Rep.* **6**, 38595; doi: 10.1038/srep38595 (2016).

**Publisher's note:** Springer Nature remains neutral with regard to jurisdictional claims in published maps and institutional affiliations.

## Supplementary Material

Supporting Information

## Figures and Tables

**Table 1:** Values for porosity, skeletal and composite density, composite specific heat capacity, and skeletal and composite thermal conductivity of the 3D AAO membrane.

Porosity P (%)	Skeletal density ρ_AAO_ (g·cm^−3^)	Composite Specific heat C_pcom_ (J·g^−1^·K^−1^)	Composite density ρ_com_ (g·cm^−3^)	Composite thermal conductivity ĸ_com_ (W·m^−1^·K^−1^)	Calculated Skeletal thermal conductivity ĸ_AAO_ (W·m^−1^·K^−1^)
40 ± 6	2.770 ± 0.054	0.885 ± 0.025	1.662 ± 0.169	0.38 ± 0.06	1.25 ± 0.25

**Table 2:** Values used for the calculation of the skeletal thermal conductivity of the 3D AAO membrane and the overall porosity along with the information source.

*P*_||_ (%)	*κ*_*air*_ (W·m^−1^·K^−1^)	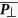 (%)	*L*_*aaoTop*_ (*μm*)	*L*_*channel*_ (*nm*)	*L*_*interchan*_ (*nm*)	N	*L*_*T*_ (*μm*)
31.8	0.026	45.9	1.2	30	230	120	33.4
SEM	Tabulated	Calculated	SEM	SEM	SEM	Theoretical	SEM

**Table 3:** Different values used to extract the cobalt and bismuth telluride thermal conductivities.

Sample	*P*_||_ (%)	*κ*_*AAO*_(W·m^−1^·K^−1^)	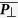 (%)	*L*_*aaoTop*_ (*μm*)	*L*_*channel*_ (*nm*)	*L*_*interchan*_ (*nm*)	N	*L*_*T*_ (*μm*)
Co	70	1.25	16	1.2	30	230	107	30
Bi_2_Te_3_	71	1.25	14	1.2	30	230	107	30
