# Supplementary material for: Tailoring thermal conductivity via three-dimensional porous alumina
Source: Sci Rep. 2016 Dec 9;6:38595. doi: 10.1038/srep38595 (PMC5146943; doi:10.1038/srep38595)
Supplement: Supporting Information [file srep38595-s1.pdf]

# Supporting Information:

# Tailoring thermal conductivity via three-dimensional porous alumina

*Begoña Abad, Jon Maiz, Alejandra Ruiz-Clavijo, Olga Caballero-Calero and Marisol Martín-González**

IMM-Instituto de Microelectrónica de Madrid (CNM-CSIC), Isaac Newton 8, PTM, E-28760 Tres Cantos, Madrid, Spain

*Corresponding author*: marisol@imm.cnm.csic.es*

**Fabrication of 3D AAO membranes**

The fabrication of the 3D AAO templates is based on the two step anodization process.[1] The aluminium foil used in this work is ultrapure (99.999%) aluminium foil provided by Advent Research Materials (England), thickness of 0.5 mm and diameter of 1.6 cm. In order to fabricate the 3D membranes, the aluminium foils are first cleaned and degreased by sonication with different polarity solvents (acetone, isopropanol, water and ethanol) and then electropolished at a constant voltage of 20 V in order to remove the oxide barrier layer created by the air, in a mixture of $HClO_4$:EtOH (25:75).

The first anodization process is performed in sulfuric acid electrolyte whose concentration is 0.3 M at a constant voltage of 25 V with a temperature in the vicinity of 1-2 ºC during 24 hours.

As a result of the first anodization, a not ordered porous alumina layer is formed. However, the pores at the alumina-aluminum interface are hexagonally distributed. The pores, form hemispherical concaves on the aluminum, which tend to arrange together with the pores. The first alumina layer was removed by chemical etching in an aqueous solution of $CrO_3$ + $H_3PO_4$ and a pulsed anodization process was carried out as published by Martín *et al* [2] During a typical pulse anodization process, mild Anodization (MA) and hard anodization (HA) constant voltage steps are periodically alternated [2-5]. However, Martín and co-workers while constant voltage is applied in MA steps, the current is limited to a defined value during HA steps and thereof the voltage is not kept constant. The material is grown thus at a defined current during HA periods. Then, the obtained AAO is selectively etched. As a consequence, the aluminum oxide grown

under HA step is removed selectively, so that transversal nanochannels are formed, establishing thus a 3D porous network. In this work, the length of the pulse ($t_{HA}$) is established in 2 s and the length of the MA ($t_{MA}$) steps is fixed to 180s.

## Electrodeposition of cobalt and bismuth telluride

The electrodeposition was made with a three electrode cell with a platinum mesh as counter electrode, a Ag/AgCl 3M KOH reference electrode and a working electrode. In the case of the cobalt film, the working electrode was a glass substrate evaporated with 5 nm of chromium and 150 nm of gold. In the case of the 3D structure, a 3D AAO template coated with 5 nm chromium and 150 nm of gold was used. This template was then glued with silver paint to a copper holder, and covered with varnish to define the area of the template exposed to the electrochemical bath and avoid deposition in the holder. For cobalt electrodeposition, the electrochemical bath was composed by a 0.4 M boric acid and 0.1 M cobalt sulphide heptahydrate (Sigma Aldrich). The electrochemical deposition was carried out at a controlled temperature of 25º C. The film was deposited at -0.78 V applied voltage versus Ag/AgCl for 15 hours, giving rise to a 40 μm thickness film. In the case of the 3D AAO structure, the potential was applied in pulses, which consisted in -0.8 V applied voltage versus Ag/AgCl for 1 second and 0 A for 0.1 seconds. A total of 18000 pulses were applied, that is, 5 hours of effective deposit. For bismuth telluride electrodeposition $0.9\cdot10^{-2}$ M $Bi^{3+}$, $1\cdot10^{-2}$ M $HTeO^{2+}$ and 1 M $HNO_3$ were used as the electrolyte solution. The reactants were purchased from Sigma Aldrich Bismuth pieces (99.999%), Sigma Aldrich (99.999%) Tellurium powder, and Panreac 65% nitric acid. The pulsed electrodeposition was performed between 0.015 V for 1 second as the on time pulse and an off time pulse of zero current density for 0.1 seconds. For a total of 52363 pulses, 14.5 hours of effective deposit. The temperature of the electrodeposition was fixed at 4º C.

## Morphological characterization

The pore diameter and thickness of the films has been studied by a Philips® XL305-FEG field emission scanning electron microscope (FE-SEM). The transversal channels were examined by a FE-SEM Hitachi one model SU8000 with TE detector operated at 0.5–3 kV.

## Density measurements

The measurements were carried out by Archimedes' principle with the assistance of an auxiliary liquid (ethanol) with a well-known density ($\rho_{et}$). A balance XS105DU from Mettler Toledo was employed to weigh the 3D sample in air ($W_{air}$) and in ethanol ($W_{et}$) so that the skeletal density is ultimately assessed by using the equation:

$$\rho = \frac{W_{air}}{W_{air}-W_{et}}(\rho_{et} - \rho_{air}) + \rho_{air}. \qquad \text{(S1)}$$

Several measurements were performed so that the related uncertainty to the mean value in the density is diminished, standing lower than 5%.

## Specific heat measurements

The measurements were performed with a Discovery DSC from TA instruments from -10ºC to 60ºC so that the 3D alumina did not experience any physical alteration. Some tests were carried out in order to guarantee the accuracy of the results, which finally presented an uncertainty lower than 5%.

## Porosity

The porosity analysis and the specific surface area measurement were carried out using $N_2$ adsorption isotherms (Micromeritic, ASAP 2020 MICROPORE DRY Analyzer). The porosity is calculated from the pore volume obtained from the BET experiment by means of the following equation:

$$P = \frac{V_{pores}}{V_{Total}} = \frac{V_{pores}}{V_{pores} + V_{aao}}, \qquad (S2)$$

where $V_{pores}$ is the pore volume, which is obtained from the BET experiment and the skeletal alumina volume, $V_{aao}$, is calculated from the mass, $m$, and the skeletal density, $\rho_{aao}$, of the alumina ($V_{aao} = m/\rho_{aao}$) [6].

**Thermal conductivity measurements**

The photoacoustic technique was employed to extract the thermal conductivity of the AAO empty and cobalt-filled samples along the direction parallel to the longitudinal channels of the 3D membrane. An incident modulated radiation impinges on the sample so that it is periodically heated and cooled. Therefore, the air in contact with the sample expands and contracts as a thermal piston thus, creating acoustic waves which are sensed by a microphone. The effective thermal conductivity of the sample is calculated by comparing the laser signal with the acoustic signal. The data reduction is performed by applying a multilayer model developed by Hu *et al.*[7] The experimental setup is described elsewhere [6, 8] and used to measure from thick and thin films to porous nanostructures [6, 8, 9]. In order to ensure the absorption of the laser beam, an 80 nm titanium layer was deposited via electron-beam evaporation onto the 3D sample.

**Porosity, density and specific heat**

In order to calculate the porosity of the 3D structure it is necessary to take into account both the longitudinal pores and the transversal nanochannels. The top-porosity which arises from the longitudinal pores can be calculated by the top SEM image analysis.

The overall porosity of the 3D AAO membrane is experimentally studied by BET via nitrogen sorption. The isotherm obtained indicates extensive mesoporosity for the 3D membrane with an average pore size of 35 nm, specific surface area of 1.6 $m^2$/g and a pore volume of 0.014 $cm^3$ $g^{-1}$. The average pore size is reasonable since the pore diameter of the longitudinal pores is around 40 nm and the transversal nanopores pore diameter is in the vicinity of 30 nm. These BET experiments provided a value of porosity of 40 ± 6 % for this sample. This value is coherent since it is higher than the porosity corresponding to the longitudinal channels analysed from the SEM images which results to be 31.8%.

The skeletal density of the 3D AAO membrane, $\rho_{AAO}$, resulted to be 2.770 ± 0.054 $g\cdot cm^{-3}$ which is in good agreement with an exhaustive recently study which established a value of 2.788 $g\cdot cm^{-3}$ for AAO membranes prepared under the sulfuric acid solution [6]. The composite density, $\rho_{com}$, of the 3D AAO membrane with air inside the pores can be calculated by the rule of mixtures which is given by the following equation:

$$\rho_{com} = P\rho_{air} + (1 - P)\rho_{AAO}, \qquad (S3)$$

where $P$ is the overall porosity of the sample and $\rho_{air}$ is the density of the air whose value is taken as $1.275 \cdot 10^{-3}$ $g\cdot cm^{-3}$ at room temperature (300 K). This rule yields to a composite density value of 1.662 ± 0.169 $g\cdot cm^{-3}$.

Figure 1 shows the specific heat of the sample as a function of the room temperature showing an increase when increasing the temperature. The room temperature value of 0.885 $J\cdot g^{-1}\cdot K^{-1}$ was taken for the data reduction for the 3D membrane.

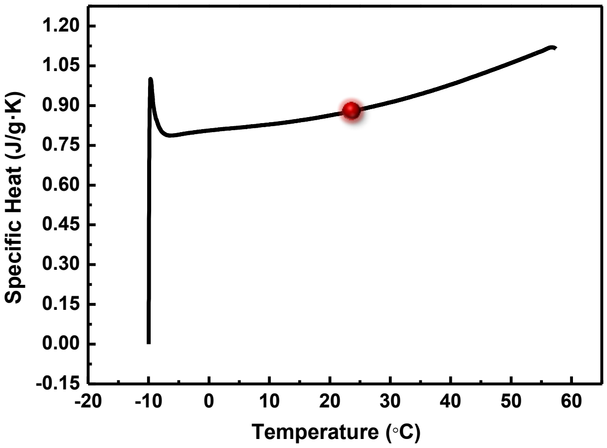


**Figure S1.** Specific heat as a function of the temperature. The red point depicts the specific value used for the thermal conductivity data reduction.

In the case of the 3D alumina membrane filled with cobalt and bismuth telluride Eq. S3 is used to calculate de the density of the composite by taking account the filler material density, and its porosity. The composite specific heat was determined by the following expression

$$C_{pcom} = \frac{P(\rho C_p)_{filler} + (1-P)(\rho C_p)_{AAO}}{\rho_{com}} \qquad \text{(S4)}$$

Where $(\rho C_p)_{filler}$ is the product of the filler material density and specific heat and $(\rho C_p)_{AAO}$ is the product of the density and specific heat of the alumina membrane. The cobalt and bismuth telluride density were and 8.86 $g \cdot cm^{-3}$ and 7.74 $g \cdot cm^{-3}$, respectively, whereas the used specific heats were 0.421 $J \cdot g^{-1} \cdot K^{-1}$ and 0.194 $J \cdot g^{-1} \cdot K^{-1}$ for cobalt and bismuth telluride, respectively [10,11].

**Uncertainty analysis**

The density, specific heat and top-porosity errors were determined as the random uncertainty within and 95% confidence interval as this error was higher than the instrumental error or other error sources:

$$x = \bar{x} \pm t_{n-1}\frac{\sigma}{\sqrt{n}}, \qquad \text{(S5)}$$

where $\bar{x}$ is the mean value of the measured property, $t_{n\text{-}1}$ is the Student-t distribution, $n$ is the number of trials and σ is the standard deviation.

The experimental error associated with the thermal conductivity measurement not only includes the random error, $E_{ran}$, but also the systematic error, $E_{sist}$, given by the lock-in amplifier and those related with the density, specific heat and thickness of the sample. The general formula used to quantify it is given by:

$$\Delta\kappa_{com} = \sqrt{(E_{ran})^2 + (E_{sist})^2 + \left(\kappa_{com}\frac{\Delta\rho}{\rho}\right)^2 + \left(\kappa_{com}\frac{\Delta C_p}{C_p}\right)^2 + \left(\kappa_{com}\frac{\Delta d}{d}\right)^2} \qquad \text{(S6)}$$

Once the $\Delta\kappa_{com}$ is obtained, $\Delta\kappa_{AAO}$ is calculated by taking into account $\Delta\kappa_{com}$ and the porosity error. The error associated with the overall porosity is given by the BET technique which for this type of sample is taken as 15% for the pore volume combined with the error corresponding with the later calculation from Eq. (S2) where the sample mass and the density are involved.

**Experimental data fitting**

Fittings of the measurements both cobalt film and 3D cobalt-filled AAO membrane are shown in Figure S2 (a) and (b) respectively.

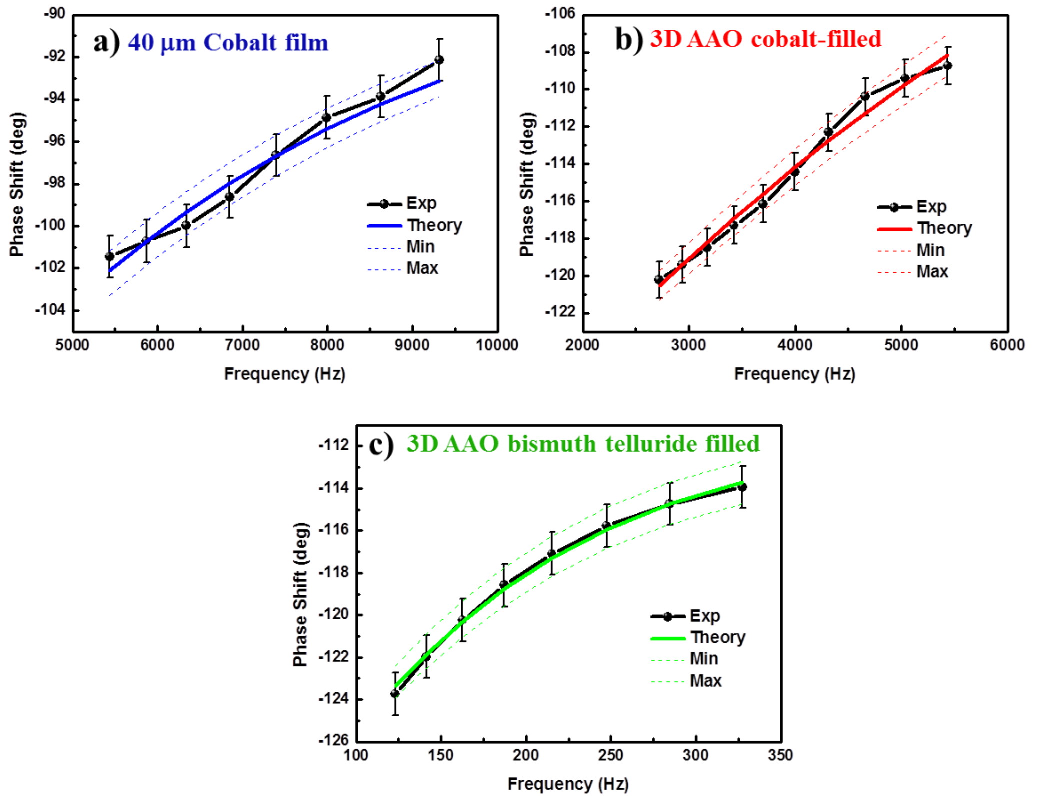


Figure S2. Experimental along with theorical fittings of (a) 40 µm cobalt film and (b) 3D cobalt-filled AAO membrane and (c) 3D bismuth telluride-filled AAO membrane.

**REFERENCES**


1. Masuda, H. et al. Metal Nanohole Arrays Made by a Two-Step Replication of Honeycomb Structures of Anodic Alumina. *Science* **268**, 1466-1468 (1995).
2. Martín, J. et al. Ordered three-dimensional interconnected nanoarchitectures in anodic porous alumina. *Nature Communications* **5,** 5130 (2014).
3. Lee, W. et al. Structural engineering of nanoporous anodic aluminium oxide by pulse anodization of aluminium. *Nature Nanotechnology* **3**, 234-239 (2008).
4. Lee, W. et al. A Continuous Process for Structurally Well-Defined Al2O3 Nanotubes Based on Pulse Anodization of Aluminum. *Nano Letters* **8**, 2155-2160 (2008).
5. Sulka, G.D. et al. Fabrication of diameter-modulated and ultrathin porous nanowires in anodic aluminum oxide templates. *Electrochimica Acta* **56**, 4972-4979 (2011).
6. Abad, B. et al. Rules to determine thermal conductivity and density of Anodic Aluminum Oxide (AAO) membranes. Journal of Physical Chemistry C **120** (10), pp. 5361-5370 (2016).
7. Hu, H. et al. Generalized theory of the photoacoustic effect in a multilayer material. *Journal of Applied Physics* **86**, 3953-3958 (1999).
8. Abad, B. et al. Thermoelectric properties of electrodeposited tellurium films and the sodium lignosulfonate effect. *Electrochimica Acta* **169**, 37-45 (2015).

9. Maiz, J. et al. Enhancement of thermoelectric efficiency of doped PCDTBT polymer films. *RSC Advances* **5**, 66687-66694 (2015).
10. Lide, D.R. *CRC Handbook of Chemistry and Physics*, 85th Edition (CRC Press LLC, 2005).
11. Bolling, G. F., Some thermal data for $Bi_2Te_3$. *The Journal of Chemical Physics* **1960,** *33*, 305-306.
